# Smile with diabetes: reflections on illness perception and diabetes management behaviors of adolescents in private health care in South Africa

**DOI:** 10.3389/fcdhc.2023.1097441

**Published:** 2023-04-28

**Authors:** Elmari Deacon

**Affiliations:** Optentia Research Programme, North-West University, Vanderbijlpark, South Africa

**Keywords:** illness perception, diabetes management behaviors, adolescence, qualitative research, positive outcomes

## Abstract

**Background:**

The association between illness perception and diabetes management has been well established in adults but is not clearly understood for adolescents. This article reflects on qualitative findings on illness perception from the perspective of adolescents, and suggests future research to operationalize findings.

**Methods:**

Qualitative document analysis was conducted on four research projects forming part of the *Smile with Diabetes* project, which aims to investigate psychosocial variables in diabetes management, including illness perception, within the adolescent and youth populations. Thematic analysis was used to derive four themes from the qualitative and review studies examined in the document analysis.

**Results:**

The voices of the adolescents were evident as four prominent themes: 1) living with diabetes leads to a sense of being different; 2) integration of diabetes into identity is critical, but difficult to achieve; 3) fear of potential negative consequences motivates adherence to treatment; 4) diabetes management is difficult, but possible.

**Conclusion:**

The findings not only confirmed the importance of illness perception in the management of diabetes by adolescents, but also indicate that illness perceptions should be investigated from a developmental perspective, specifically taking identity development into consideration in this group. Adolescents should be made aware of how their thinking about diabetes and its management affects their experience of living with diabetes and its future management. This study further contributes to the literature by focusing on the patient’s voice in understanding living with a chronic condition, and reassures that positive outcomes are possible when living with a chronic condition such as diabetes.

## Introduction

A growing body of evidence in diabetes management associates illness perception with successful control of the condition and improved health outcomes in the adult population (1, 2). Adolescents, however, generally struggle to adhere to diabetes care plans (3, 4), so that a better understanding of their perception of illness could assist in improving its control and health outcomes (5, 6). Furthermore, illness perceptions are formed during adolescence, which makes this an important period in which to engage in thinking about the condition (7, 8).

Despite strong evidence of the importance of illness perception for adults, that for adolescents is inconclusive (9, 10). Available research mostly focuses on quantitative studies that provide inconsistent results (6, 11). In this article, the qualitative findings of three studies, supported by one review study, emphasize the importance of illness perception within the developmental framework of the adolescent and suggest future research to operationalize the findings. The originality of this articles lies in the effective use and reflection on the patient’s voice, which is often absent in medical research.

### Diabetes management in adolescents

Type 1 diabetes is a condition diagnosed when the body produces insufficient insulin to function well (12, 13). When the glucose content in the blood increases, it can lead to several adverse consequences, including kidney failure, damage to sight and loss of limbs, all of which impede a person’s proper functioning (14). Diabetes management behaviors, such as monitoring blood glucose levels, administering insulin and maintaining a healthy diet aim to keep blood glucose levels within acceptable levels and prevent long-term damage (15). Adherence to a diabetes care plan prescribed by a medical professional requires continuous inputs from the patient and makes demands that often lead to psychological challenges, such as stress, anxiety and depression (16, 17). Thus, managing diabetes is not only reliant on medical interventions (18), but involves psychological aspects, such as perception of the illness by the patient, that also need to be addressed (19).

### Illness perception

Illness perception can be defined as a set of beliefs about an illness in terms of five dimensions, namely, identity (symptoms attributed to the illness and illness label); timeline (expected course of the illness and duration of its symptoms); consequences (beliefs regarding short- and long-term impact of the illness on the physical, psychological and social well-being of the patient and others); cause (factors perceived to be responsible for the illness); and control/cure (beliefs about illness management and controllability) (20–22). The Common Sense Model (CSM) of illness perception is a self-regulatory approach that proposes that individuals develop their own set of beliefs (illness representations) about their condition, which is based on their understanding or experience of the condition and provides a “framework of action” influencing coping strategies and action plans, for example self-management behaviors or adherence to treatment care plans (23).

The CSM has been operationalized in the Illness Perception Questionanire (IPQ, IPQ-R and BIPQ) (2) and has been used in quantitative studies that investigated the association between illness perception and glycemic control for adults (2). However, in adolescents, this association is not clear (24). In a review by Law and colleagues (10), the illness representations of identity, timeline, treatment control and consequences were the most frequently investigated domains across studies, but the relationships among each of these domains and self-management were found to be variable with most studies suggesting that illness identity, timeline and consequences had no significant relationship with self-management (10). As for treatment control, adolescents who believe that their illness can be controlled or managed by treatments were more likely to report performing self-management behaviors, which is consistent with the CSM model. However, there was no association evident between self-management and personal control, which could be explained by the transitional nature of diabetes management during the time of adolescence (10).

Any difference in associations of illness perception in adults and adolescents can be explained by a closer look at the developmental perspective. Chaing and colleagues (25) explained development-specific tasks for adolescents living with diabetes. Some of the aspects relevant to this study include that normal developmental issues for early adolescents are to form a strong sense of self-identity, while family issues in type 1 diabetes management is the renegotiation of the parents’ and teenager’s roles in diabetes management to be acceptable to both. For later adolescents, normal developmental issues include establishing a sense of identity after high school and integrating the illness into a new lifestyle, which also provides an opportunity for the emergence of an identity that incorporates diabetes, as well as illness perceptions that support diabetes management behaviours (25).

The focus of this article is on illness perception from the perspective of the adolescents. The conclusions drawn here fill the gap in the literature on diabetes management and illness perception of adolescents by emphasizing the importance of the latter within the developmental framework of young people, as well as the importance of one of the most underutilized and relatively ignored resources in diabetes care, namely the voice of the patient.

## Materials and methods

### Study design

In this study, qualitative document analysis was conducted to illustrate the illness perception of adolescents in private health care in South Africa. By collecting information from different research reports, findings across studies can be corroborated to reduce the bias in a single investigation (26). Four studies from the *SMILE with Diabetes* project were analyzed and reflected upon to emphasize the patents’ voice and to indicate future suggestions for research. The *SMILE with Diabetes* project started in 2015, with the principal researcher being the author, Elmarí Deacon, who is affiliated with the Optentia Research Unit at North-West University. This research project aims to investigate the psycho-social variables in adjusting to diabetes management behaviors in adolescents and young adults. The project’s focus is on understanding the patients’ voice in terms of social support (S), meaning-making (M) and illness perception (L), and to use findings from studies to develop interventions (I) to assist others to also achieve effective diabetes management (E). Since the first approvals for the project, 13 studies have been completed with another six in process.

Ethical clearance for the main project was granted by the Humanities and Health Research Ethics Committee (HHREC) of the North-West University Institutional Research Ethics Regulatory Committee (NWU-IRERC) NWU-HS-2015-0111.

### Data collection and analysis

#### Data collection

Four studies were used as data sources. They were either reviews or qualitative in nature, using semi-structured interviews.

##### Document 1 (Jonker, Deacon, Van Rensburg & Segal, 2018)

This article aimed to explore the illness perceptions of adolescents with well-controlled type 1 diabetes mellitus (T1DM) in South Africa. Semi-structured interviews with nine adolescents were conducted and the following themes emerged: Living with diabetes becomes a way of life; managing diabetes leads to being different; acknowledgment of potential negative health consequences; diabetes is manageable; procurement of adequate knowledge; embracing accountability to comply with the specific management protocol and acceptance of lot/fate. These perceptions contributed to adherence to diabetes care plans and should be explored and developed among adolescents with TIDM to improve their management of the condition.

##### Document 2 (Lesage, Deacon, Van Rensburg & Segal, 2021)

This article explored the illness perception of adolescents living with uncontrolled type 1 diabetes (T1D) and how these perceptions interacted with its management. A qualitative, explorative design with eight semi-structured interviews was used to collect data, from which the following themes were derived: illness perception of T1D is negative; living with T1D leads to a sense of being different; management of T1D is challenging; and management of T1D is motivated by fear. These results may give additional insights into the awareness of illness perception when designing successful interventions.

##### Document 3 (Williams, Deacon, Van Rensburg & Segal, 2023)

In this article, a qualitative research approach using semi-structured online interviews was used to gather data on the experience of adolescents living with T1D (mean age 14.9 years) using continuous glucose monitoring (CGM). Thematic analysis was used to evaluate seven interviews. Themes emerging from the data indicated that CGM creates a sense of control over diabetes management; that it assists in incorporating diabetes management into their identity; and that CGM creates opportunities for positive outcomes. Despite the users’ awareness of being different by being subject to diabetes, CGM assisted in creating a sense of belonging and feeling of empowerment to take responsibility for management of the condition. This awareness of control empowered the study population to be more responsible with their diabetes management, contributing to achieving a better quality of life.

##### Document 4 (Harmse, 2020)

This mini-dissertation aimed to perform a critical review to evaluate the scientific literature regarding identity development in adolescents living with T1DM, to gain new knowledge regarding the chronic condition and how an adolescent develops an identity while living with it. The following themes emerged: identity develops differently in adolescents living with T1DM from young people who do not have it; adolescents living with type 1 diabetes can either incorporate or contain his or her diabetes in relation to their identity development; and external factors influence identity development in adolescents living with T1DM. These findings emphasize the significant need that exists for research in identity development in adolescents living with T1DM. Furthermore, interventions for adolescents to help them establish an identity while living with a chronic condition such as T1DM are also needed.

#### Data analysis

The documents were analyzed thematically, taking the following steps (27): immersion in the data to become familiar with its scope, developing initial codes, searching for and reviewing themes, naming and defining themes and, finally, reporting the outcomes used to identify themes. Steps to ensure methodological rigour and trustworthiness in each document included bracketing and reflexivity, as well as the use of a co-coder. The researchers in all the studies were adequately trained to gather and analyse the data. The themes as identified in the documents were used as the data in the current manuscript. As the aim of the current study was to reflect on illness perception from the perspective of the adolescent, an insider perspective was used, with critical reflection as part of the analysis process. The following steps were incorporated in the process (28): bracketing was implemented throughout the research process to enhance the trustworthiness of the study. Credibility was achieved through researcher reflexivity and peer discussions with other researchers familiar with the studies. Findings were not derived from biased interpretations or assumptions made by the authors, thus promoting confirmability. Transferability was enhanced by providing contextual information on the documents to form a better understanding of their settings.

## Results

Four themes emerged from the data: 1) Living with diabetes leads to a sense of being different; 2) integration of diabetes into one’s identity is critical, but difficult to achieve; 3) fear of potential negative consequences motivates adherence to treatment; 4) diabetes management is difficult, but possible.

### Theme 1: living with diabetes leads to a sense of being different

The most prominent theme from the document analysis was that living with diabetes left participants feeling being different from their peers (6, 29). This difference was accounted for in both the patient’s voice and the critical review. Adolescents living with well-controlled and uncontrolled diabetes both reported, “*I am not similar to other people who have a normal pancreas,” (* 29 *)* and *“I kinda feel like the odd one out,” (* 6 *)* while the review reported that identity develops differently in adolescents living with type 1 diabetes mellitus from those who do not have the condition (8). This difference was mostly a result of the self-management behaviors that they had to perform. Fitting in and belonging to a peer group is an important need in adolescence (25), so that feeling different from one’s peers could impact on identity development and potentially result in emotional distress.

All participants actively sought situations in which they could feel “normal” and belong to a group, such as a sports team (6, 29, 30). For adolescents using CGM, being different and wearing a CGM device also provided opportunities to reach out to others using the equipment and creating a feeling of belonging; as one participants noted, she *“gets excited”* when she sees someone else wearing a CGM (30).

### Theme 2: integration of diabetes into one’s identity is critical, but difficult to achieve

Most documents analyzed reported on the impact of living with diabetes on the identity and identity development of adolescents. According to the critical review, adolescents living with type 1 diabetes mellitus can either incorporate or contain their chronic condition in relation to their identity development (8). The challenge was evident from the qualitative studies with quotes of those living with well-controlled diabetes elaborating on how they managed to incorporate diabetes into their identity and experienced that living with diabetes becomes a way of life (*“something that is part of me” (* 29 *)*), whereas those who did not manage diabetes well had a negative perception of the illness (*“it kinda sucks” (* 6 *)*) and used avoidance strategies to manage their condition (*“I don’t do it because it is too much work and it’s too much pressure on me” (* 6 *)*).

It is important to note that *CGM* assists in incorporating diabetes management into one’s identity (*“made it seem less like a disability” (* 30 *)*) and hence can be seen as a medical intervention not only to improve psychical outcomes but also assisting in identity integration as CGM became an integral *“part of myself” (* 30 *)* and the participant’s way of life.

Identity development is a crucial and complicated task to be accomplished during adolescence; successfully completing this achievement by those living with diabetes is especially challenging. Although most available research reports on this challenge, it is important to emphasize that in two of the studies consulted, incorporation of diabetes into the identity of the adolescent was possible, resulting in a new normal way of life.

### Theme 3: fear of potential negative consequences motivates adherence to treatment

Accounts of beliefs about consequences of not managing diabetes well varied in the documents studied. Adolescents living with well-controlled diabetes acknowledged potential adverse health consequences (29), whereas for the uncontrolled group the management of T1D was motivated by fear (6). The undertone of fear as a motivating factor in these studies should not be overlooked, but the effect of fear on adherence to the diabetes care plan and incorporating diabetes management behaviors differed. Fear spurred those living with well-controlled diabetes to action: “*If I don’t manage it properly it could kill me in the end.” (* 29 *)* These adolescents were motivated to comply with expected diabetes management behaviors and as a result achieve good health outcomes, whereas those experiencing the uncontrolled condition were overwhelmed by fear and accepted the negative consequence as given, which resulted in their not changing their ways: *“I know what’s going to happen in a few years, I feel fine now, but I know what’s coming.” (* 6 *)*

### Theme 4: diabetes management is difficult, but possible

Perceptions of the manageability of diabetes varied significantly among groups. Adolescents living with well-controlled diabetes believed that diabetes is manageable (*“It is not the most difficult thing to manage” (* 29 *)*), whereas those reconciled to uncontrolled diabetes averred that management of T1D is challenging (“*It’s kinda difficult” (* 6 *)*). Additionally, adolescents using CGM experienced that it creates a sense of control over diabetes management (*“because now I can constantly see my levels” (* 30 *)*), which influenced their perception of the manageability of diabetes. The perception that diabetes is manageable developed over time and was affected by the actions these young people took (see theme 3) to empower them to manage their condition.

Diabetes management behaviors are well known as regular blood glucose monitoring, administration of insulin and a healthy diet, but adolescents living with well-controlled type 1 diabetes (29) reports on additional tasks in diabetes management, including procurement of adequate knowledge, embracing accountability to comply with specific management protocols, and accepting their fate, which assisted this specific group in managing diabetes well.

It was noteworthy that all participants in the studies reported on the unpredictability of diabetes and how it requires different actions to be taken; but all those with the condition well-controlled and those using CGM still believed that diabetes is manageable. Where differences of opinion existed, they could be explained by adolescents lacking control giving up because they felt that they could not manage their situation, whereas those exercising good control and using CGM were empowered by their sense of achievement and even experiencing positive outcomes, such as growth (30). These themes are summarized in **Table 1**.

**Table 1:** Summary of themes.

Theme	Source
Living with diabetes leads to a sense of being different	Managing diabetes leads to being different (doc 1) Living with T1D leads to a sense of being different (doc 2) CGM creates opportunities for positive outcomes (belonging) (doc 3) Identity develops differently in adolescents living with T1D than those who do not have a chronic condition (doc 4)
Integration of diabetes into one’s own identity is critical, but difficult to achieve	Living with diabetes becomes a way of life (doc 1) Illness perception is negative (doc 2) CGM assists in incorporating diabetes management into their identity (doc 3) Adolescents living with T1D can either incorporate or contain their condition in relation to their identity development (doc 4)
Fear of potential negative consequences motivates adherence to treatment	Acknowledging of potential negative health consequences (doc 1) Management of T1D is motivated by fear (doc 2)
Diabetes management is difficult, but possible	Diabetes is manageable (doc 1) (Management tasks: Procurement of knowledge; Embracing accountability to comply with the specific management protocol; Acceptance of lot/fate (doc 1)) Management of T1D is challenging (doc 2) CGM creates a sense of control over diabetes management (doc 3)

## Discussion

In this qualitative document analysis on illness perception and diabetes management, I have highlighted the influence of identity development in adjusting to diabetes management behaviors. The importance of identity development in this group is emphasized by the first two themes, which relate to a person’s sense of being different due to living with diabetes and its integration into their identity. This is not surprising as identity development and the management of peer pressure are two of the main tasks at this developmental stage (31). For adolescents living with diabetes, the challenge is clear: even though they strive to belong and to fit in with their peers, living with their illness makes them different from others; whereas making the condition and its management part of their being adds much responsibility to their already complex lives. Adjusting to diabetes happens over time, just as developing an identity does. Oris and colleagues (32) identified four phases of identity development, which was evident in the adolescent participants in the various studies reviewed here: engulfment was evident in all groups as they reported being overwhelmed by the initial diagnosis (6, 29, 30); rejection of diabetes as part of their lives was evident in the uncontrolled group (6); acceptance was seen in the well-controlled and CGM groups (29, 30); and enrichment was seen also in the well-controlled and CGM groups (29, 30). The differences in identity development among the well-controlled and uncontrolled groups imply that teaching adolescents to think more like the well-controlled group could be beneficial, and that the use of CGM empowers not only diabetes management but potentially also identity development in adolescents living with type 1 diabetes.

The important role of illness perception in the successful management of diabetes was highlighted through the expression of participants clearly supporting the self-regulatory framework suggested by the CSM model (23). Leventhal posits that the set of beliefs about a condition provides a framework of action influencing adherence to treatment plans, which was evident in the third and fourth themes. Adolescents living with well-controlled diabetes believed diabetes management was possible, and that adhering to diabetes care plans could prevent adverse consequences of the condition. This motivated them to go beyond the basic diabetes management tasks, and further procured knowledge and accepted accountability for the management of their illness. This is in contrast to the adolescents who believed that diabetes management was impossible to achieve, which left them fearful of the negative consequences of their condition and using coping strategies such as avoidance of management behaviors. The interaction between themes is further explained by the proposed extended model of illness self-regulation (18), which states that attitudes towards illness are activated by the presentation of health-threatening stimuli (theme 2) and that behavioral and treatment beliefs are determinants of coping procedures (theme 3). As adolescence is a stage at which cognitive development takes place (33), it is imperative to hold discussions with this group of patients on their beliefs about their condition, as well how this influences the success they achieve with diabetes management. As this is the phase in which perceptions, beliefs and habits regarding diabetes are formed (34), it would be of great benefit to these patients to also see a mental healthcare practitioner during this developmental phase.

Although the aim of this study was not to represent the dimensions of illness perception according to the CSM, the consequences and control dimensions of illness perception were highlighted in the themes presented by the studies. The effect of beliefs about the consequences of diabetes, as well as beliefs in its control, underlaid themes 3 and 4 and the discussion above. The differences in personal control are important to note: those who managed diabetes well and those using CGM were empowered by positive feedback, which gave them confidence in managing the condition (29, 30), even when they struggled. Adolescents in these groups held strong beliefs about personal control, although the control of treatment may not have been effective. A practical example of this is the difference in the beliefs around the unpredictability of diabetes, as well as the consequences of not managing the condition well. Although all the groups experienced blood glucose sometimes not responding to treatment, as would have been expected, the well-controlled group still saw diabetes as manageable, and those on CGM reported a sense of control of their condition. In the well-controlled group, the potential negative consequences of diabetes motivated them to acquire knowledge, in contrast to the uncontrolled group not taking action.

Living with diabetes has been described as a burden, resulting is many psychological challenges, but positive outcomes were possible in the studies examined. The group of adolescents living with well-controlled diabetes were empowered by living well, thus gaining confidence in themselves (29), while those using CGM declared that this action creates opportunities for raising awareness and experiencing a feeling of belonging (30). This reflects a growing body of evidence reporting the possibility of positive outcomes and meaning making in living with a chronic condition, such as type 1 diabetes (35). The study of positive psychology focuses on happiness, meaning-making and character strengths (36), and many positive psychology interventions have been developed for those living with diabetes (37). This study emphasizes the need for these interventions, notably in respect of identity development and illness perception in the vulnerable adolescent group.

## Conclusion: strengths and limitations

The aim of this study was to reflect on findings from previous research on illness perception held by adolescents living with diabetes. The themes identified emphasized the important role of illness perception in diabetes management, especially through the voice of the patient. Furthermore, the developmental stage of adolescence was highlighted as a critical context to consider in diabetes management interventions, and the potential of positive outcomes was also evident. The relevance of these findings in the African context are far-reaching as the incidence of diabetes is increasing beyond expectation, while national healthcare systems lack the capacity for managing the condition (38). Within these struggling circumstances, often failing the patient, the development of interventions to assist those living with diabetes to control the way they see the condition would make a valuable contribution to their lived experiences. Although the integration of identity development and illness perception in adolescents is an important contribution in the treatment of diabetes, the applicability of this process needs to be further investigated in different populations. The documents analyzed in the current study was representative only of private healthcare patients in South Africa, yet the majority of adolescents living with diabetes have to make do with the public healthcare system. Cultural differences in illness perceptions (39) also need to be explored further, and studies on more diverse and bigger populations are required.

## Data availability statement

The original contributions presented in the study are included in the article/supplementary material. Further inquiries can be directed to the corresponding author/s.

## Author contributions

The author confirms sole responsibility for the study conception and design, data collection, analysis and interpretation of results, and manuscript preparation.

## References

[1] Harvey JN Lawson VL. The importance of health belief models in determining self-care behaviour in diabetes. Diabetes Med (2009) 26(1):5–13. doi: 10.1111/j.1464-5491.2008.02628.x 19125754

[2] McSharry J Moss-Morris R Kendrick T. Illness perceptions and glycaemic control in diabetes: a systematic review with meta-analysis. Diabetic Med (2011) 28(11):1300–10. doi: 10.1111/j.1464-5491.2011.03298.x 21418098

[3] Bell T Noar SM Shafer A. The process of developing and pretesting narrative messages for adolescents with type 1 diabetes. J Communication Healthcare (2022) 15(2):102–11. doi: 10.1080/17538068.2021.2018154

[4] McGrady ME Peugh JL Hood KK. Illness representations predict adherence in adolescents and young adults with type 1 diabetes. Psychol Health (2014) 29(9):985–98. doi: 10.1080/08870446.2014.899361 24588345

[5] Lawson VL Bundy C Harvey JN. The development of personal models of diabetes in the first 2 years after diagnosis: a prospective longitudinal study. Diabetic Med (2008) 25(4):482–90. doi: 10.1111/j.1464-5491.2008.02394.x 18341594

[6] Lesage S Deacon E Van Rensburg E Segal D. ‘It kinda sucks’: illness perception of a group of south African adolescents with type 1 diabetes mellitus. Afr J Primary Health Care Family Med (2021) 13(1):2782. doi: 10.4102/phcfm.v13i1.2782 PMC8007989 33764139

[7] Verschueren M Oris L Claes L Moons P Weets I Luyckx K. Identity formation in adolescents and emerging adults with type 1 diabetes. Psychol Health Med (2020) 25(5):519–29. doi: 10.1080/13548506.2019.1653482 31469304

[8] Harmse E. Exploring identity development in adolescents living with type 1 diabetes: a critical review. South-Africa: North-West University (2020).

[9] Griva K Myers LB Newman S. Illness perceptions and self efficacy beliefs in adolescents and young adults with insulin dependent diabetes mellitus. Psychol Health (2000) 15(6):733–50. doi: 10.1080/08870440008405578

[10] Law GU Tolgyesi CS Howard RA. Illness beliefs and self-management in children and young people with chronic illness: a systematic review. Health Psychol Rev (2014) 8(3):362–80. doi: 10.1080/17437199.2012.747123 25053219

[11] Paduraru AE Soponaru C. Illness representation among adolescents: a qualitative approach. Revista de Cercetare si Interventie Sociala (2020) 71:438–52. doi: 10.33788/rcis.71.26

[12] Magliano DJ Zimmet P Shaw JE. Classification of diabetes mellitus and other categories of glucose intolerance. In: DeFronzo RA Ferrannini E Zimmet P Alberti KGM, editors. International textbook of diabetes mellitus, 4th ed. Chichester, England: Wiley Blackwell (2015). p. 3–16.

[13] Sen S Chakraborty R De B. Diabetes mellitus in 21st century. Puchong, Selangor D. E: Springer Singapore (2018).

[14] Forbes JM Harcourt B. Pathogenesis of diabetic microvascular complications. In: DeFronzo RA Ferrannini E Zimmet P Alberti KGM, editors. International textbook of diabetes mellitus, 4th ed. Chichester, England: Wiley Blackwell (2015). p. 873–88.

[15] Woods J Peters A. The basics of type 1 diabetes. In: Peters A Wood J Zacharatos MZ, editors. The type 1 diabetes self-care manual (Arlington, VA: American Diabetes Association) (2018). p. 1–25.

[16] Hagger V Trawley S Hendrieckx C Browne JL Cameron F Pouwer F. Diabetes MILES youth–Australia: methods and sample characteristics of a national survey of the psychological aspects of living with type 1 diabetes in Australian youth and their parents. BMC Psychol (2016) 4(1):1–3. doi: 10.1186/s40359-016-0149-9 27519408 PMC4983064

[17] Hunter CM. Understanding diabetes and the role of psychology in its prevention and treatment. Am Psychol (2016) 71(7):515–25. doi: 10.1037/a0040344 27690481

[18] Hagger MS Orbell S. The common sense model of illness self-regulation: a conceptual review and proposed extended model. Health Psychol Rev (2021) 16(3):347–77. doi: 10.1080/17437199.2021.1878050 33461402

[19] Broadbent E Donkin L Stroh JC. Illness and treatment perceptions are associated with adherence to medications, diet, and exercise in diabetic patients. Diabetes Care (2011) 34(2):338–40. doi: 10.2337/dc10-1779 PMC3024345 21270191

[20] Petrie KJ Jago LA Devcich DA. The role of illness perceptions in patients with medical conditions. Curr Opin Psychiatry (2007) 20(2):163–7. doi: 10.1097/YCO.0b013e328014a871 17278916

[21] Petrie KJ Weinman J. Perceptions of health and illness: current research and applications. London: Harwood Academic Publishers (1997).

[22] Petrie KJ Weinman J. Patients’ perceptions of their illness: the dynamo of volition in health care. Curr Dir Psychol Sci (2012) 21(1):60–5. doi: 10.1177/0963721411429456

[23] Leventhal EA Patrick-Miller L Robitaille C. Illness representations: theoretical foundations. In: Petrie KJ Weinman JA, editors. Perceptions of health and illness: current research and applications. Amsterdam: Harwood Academic (1998). p. 19–45.

[24] Prinsloo NM. Illness perception in adolescents with controlled and uncontrolled diabetes: a rapid review. South-Africa: North-West University (2017).

[25] Chiang JL Kirkman MS Laffel LM Peters AL. Type 1 diabetes sourcebook authors. type 1 diabetes through the life span: a position statement of the American diabetes association. Diabetes Care (2014) 37(7):2034–54. doi: 10.2337/dc14-1140 PMC5865481 24935775

[26] Bowen GA. Document analysis as a qualitative research method. Qual Res J (2009) 9(2):27–40. doi: 10.3316/QRJ0902027

[27] Braun V Clarke V Hayfield N Terry G. Thematic analysis. handbook of research methods in health social sciences. (2018). Available at: https://search-ebscohostcom.nwulib.nwu.ac.za/login.aspx?direct=true&db=edssjb&AN=edssjb.978.981.10.5251.4.103&site=eds-live.

[28] Lincoln YS Guba EA. Naturalistic inquiry. Beverly Hills: SAGE (2021).

[29] Jonker D Deacon E Van Rensburg E Segal D. Illness perception of adolescents with well-controlled type 1 diabetes. Health Psychol Open (2018) 1–9. doi: 10.1177/2055102918799968 PMC6146331 30245842

[30] Williams L Deacon E Van Rensburg E Segal D. Continuous glucose monitoring empowers adolescents to take responsibility of diabetes management. Afr J Primary Health Care Family Med (2023) 15(1):a3879. doi: 10.4102/phcfm.v15i1.3879 PMC10157418 37042539

[31] Chao AM Minges KE Park C Dumser S Murphy KM Grey M. General life and diabetes-related stressors in early adolescents with type 1 diabetes. J Pediatr Health Care (2016) 30(2):133–42. doi: 10.1016/j.pedhc.2015.06.005 PMC4733440 26234658

[32] Oris L Rassart J Prikken S Verschueren M Goubert L Moons P. Illness identity in adolescents and emerging adults with type 1 diabetes: introducing the illness identity questionnaire. Diabetes Care (2016) 39(5):757–63. doi: 10.2337/dc15-2559 26989179

[33] Cosma A Băban A. Emotional responses of adolescents with type 1 diabetes: the role of illness representations and coping. Cognition Brain Behavior Interdiscip J (2017) 21(2):117–. doi: 10.24193/cbb.2017.21.08

[34] Fortenberry KT Berg CA King PS Stump T Butler JM Pham PK. Longitudinal trajectories of illness perceptions among adolescents with type 1 diabetes. J Pediatr Psychol (2014) 39(7):687–96. doi: 10.1093/jpepsy/jsu043 PMC4161993 24934247

[35] Kruger S Deacon E van Rensburg E Segal DG. Young adult women’s meaning-making of living with type 1 diabetes: towards growth and optimism. Psychol Health (2021), 1–8. doi: 10.1080/08870446.2021.1977303 34510968

[36] Sin NL Lyubomirsky S. Enhancing well-being and alleviating depressive symptoms with positive psychology interventions: a practice-friendly meta-analysis. J Clin Psychol (2009) 65(5):467–87. doi: 10.1002/jclp.20593 19301241

[37] Jaser SS Datye K Morrow T Sinisterra M LeStourgeon L Abadula F. THR1VE! positive psychology intervention to treat diabetes distress in teens with type 1 diabetes: rationale and trial design. Contemp Clin Trials (2020) 96:106086. doi: 10.1016/j.cct.2020.106086 32682996 PMC7494522

[38] Nuche-Berenguer B Kupfer LE. Readiness of sub-saharan africa healthcare systems for the new pandemic, diabetes: a systematic review. J Diabetes Res (2018) 2018:1–12. doi: 10.1155/2018/9262395 PMC5835275 29670916

[39] Abubakari AR Jones MC Lauder W Kirk A Anderson J Devendra D. Associations between knowledge, illness perceptions, self-management and metaboliccontrol of type 2 diabetes among African and European-origin patients. J Nurs Healthcare Chronic Illness (2011) 3(3):245–56. doi: 10.1111/j.1752-9824.2011.01098.x

